# T Cell Subsets in Graft Versus Host Disease and Graft Versus Tumor

**DOI:** 10.3389/fimmu.2021.761448

**Published:** 2021-10-05

**Authors:** Hua Jiang, Denggang Fu, Alan Bidgoli, Sophie Paczesny

**Affiliations:** Department of Microbiology and Immunology and Pediatrics, Medical University of South Carolina, Charleston, SC, United States

**Keywords:** graft versus host disease, graft versus tumor, tissue resident memory t cell, cell therapy, t cells subsets

## Abstract

Allogeneic hematopoietic cell transplantation (allo-HCT) is an essential therapeutic modality for patients with hematological malignancies and other blood disorders. Unfortunately, acute graft-versus-host disease (aGVHD) remains a major source of morbidity and mortality following allo-HCT, which limits its use in a broader spectrum of patients. Chronic graft-versus-host disease (cGVHD) also remains the most common long-term complication of allo-HCT, occurring in reportedly 30-70% of patients surviving more than 100 days. Chronic GVHD is also the leading cause of non-relapse mortality (NRM) occurring more than 2 years after HCT for malignant disease. Graft versus tumor (GVT) is a major component of the overall beneficial effects of allogeneic HCT in the treatment of hematological malignancies. Better understanding of GVHD pathogenesis is important to identify new therapeutic targets for GVHD prevention and therapy. Emerging data suggest opposing roles for different T cell subsets, e.g., IFN-γ producing CD4+ and CD8+ T cells (Th1 and Tc1), IL-4 producing T cells (Th2 and Tc2), IL-17 producing T cells (Th17 and Tc17), IL-9 producing T cells (Th9 and Tc9), IL-22 producing T cells (Th22), T follicular helper cells (Tfh), regulatory T-cells (Treg) and tissue resident memory T cells (Trm) in GVHD and GVT etiology. In this review, we first summarize the general description of the cytokine signals that promote the differentiation of T cell subsets and the roles of these T cell subsets in the pathogenesis of GVHD. Next, we extensively explore preclinical findings of T cell subsets in both GVHD/GVT animal models and humans. Finally, we address recent findings about the roles of T-cell subsets in clinical GVHD and current strategies to modulate T-cell differentiation for treating and preventing GVHD in patients. Further exploring and outlining the immune biology of T-cell differentiation in GVHD that will provide more therapeutic options for maintaining success of allo-HCT.

## Introduction

Allogeneic hematopoietic cell transplant (allo-HCT) is a remarkably successful immunotherapy in large part due to the graft-versus-tumor (GVT) effect. Unfortunately, GVT is tethered to the pathogenesis of acute graft versus host disease (aGVHD). The detailed pathogenesis of acute GVHD (aGVHD) has recently been reviewed in depth (1, 2). Overall, T cells are indispensable mediators of aGVHD pathogenesis since GVHD rarely develops after syngeneic or T cell-depleted transplants (3–7). Both aGVHD and GVT have been found to be initiated by antigen presenting cells (APCs) derived from the donor and from host activating donor T cells (8, 9). Such activation leads to the release of inflammatory cytokines, with subsequent proliferation of alloreactive T cells, resulting in host damage and further inflammation. Around 15-20% of hematopoietic cell transplant patients develop severe refractory GVHD leading to mortality (10, 11). Chronic GVHD (cGVHD) pathogenesis is a complex process involving both B and T cells (12). The process was reviewed recently in detail (1). Essentially, crosstalk between B and T cells leading to the proliferation of germinal centers allowing the production of allo-reactive antibodies appears to be the overlying process of the disease. The mainstays of GVHD prevention include anti-thymocyte globulin, calcineurin inhibitors and post-transplant cyclophosphamide, and first line therapies include corticosteroids in addition to calcineurin inhibitors. However, treatment with these drugs negatively affect desirable GVT (13). In addition, steroid-refractory GVHD (SR-aGVHD) patients have dismal outcomes, thus representing an urgent need for developing new treatment strategies in the field of transplant medicine (14). That said, recent breakthroughs have been made including the positive result of the randomized phase III clinical trial evaluating ruxolitinib versus best available treatment (BAT) in SR-aGVHD (15). Similarly, positive results were seen in SR-cGVHD comparing ruxolitinib versus BAT (16). The central role of T cells in the pathogenesis of GVHD has also led to extensive studies in manipulating T cell populations to reduce GVHD severity. Specific T cell subsets have been found to either exacerbate or alleviate GVHD/GVT, a finding that is currently being exploited in novel treatment options in preclinical and/or clinical studies.

## T Cells Inducers of GVHD

**Figure 1:**
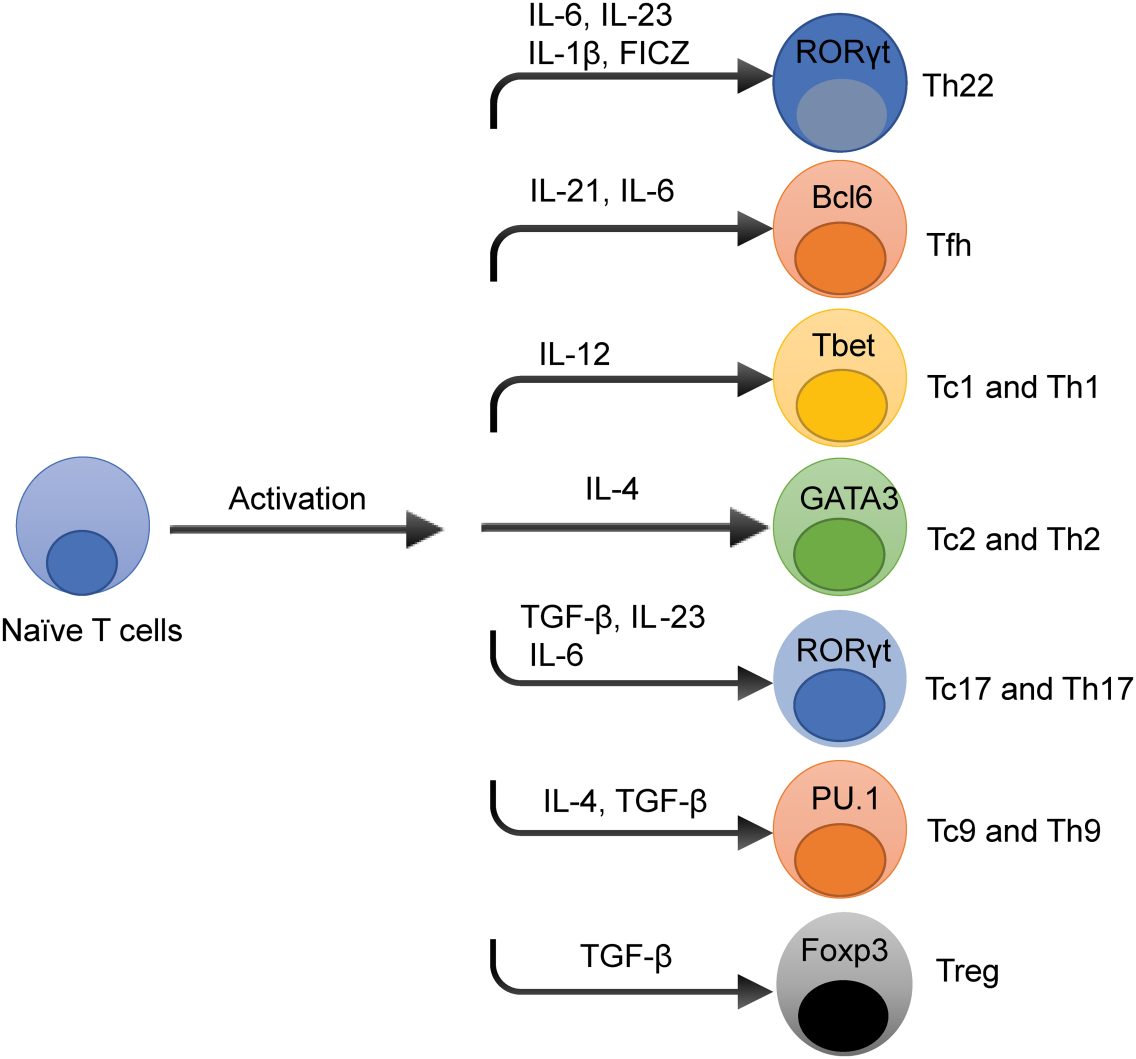
Overview of T Cells Differentiation Pathways. The cytokine and transcription factors (TFs) niche dictates T cell differentiation in spite of the stimulation of T cell receptor signaling pathways. The prototypical cytokines and TFs that regulate each T cell subset differentiation fate are depicted. These cytokines and TFs that influence T cell differentiation have effects on the development of multiple T cell subsets, such as interleukin-6 (IL-6) is essential for T helper type 22 (Th22), T follicular helper (Tfh), and T helper 17 (Th17) cell development.

T cells differentiation is initiated when naïve T cells are stimulated by antigens in the presence of MHC molecules under a particular milieu of cytokines their corresponding signaling pathways to develop into different T cell subsets that acquire specialized effector cell phenotypes (17). As shown in **Figure 1**, these T cell subsets are characterized by the production of signature cytokines and expression of specific transcription factors (TFs). The specific cytokines and TFs are activated by signal transducer and activator of transcription (STAT) family members to confer specialized functions to the T cell subsets. These cytokines and TFs that regulate T cells differentiation may have effects on the development of multiple T cell subsets. For example, interleukin 6 (IL-6) is essential for T follicular helper (Tfh) and T helper type 17 (Th17) differentiation through the STAT5 signaling pathway (18). Different T cell subsets have been involved in several inflammatory diseases (19, 20), and may allow the development of novel treatment strategies (21).

**Figure 2:**
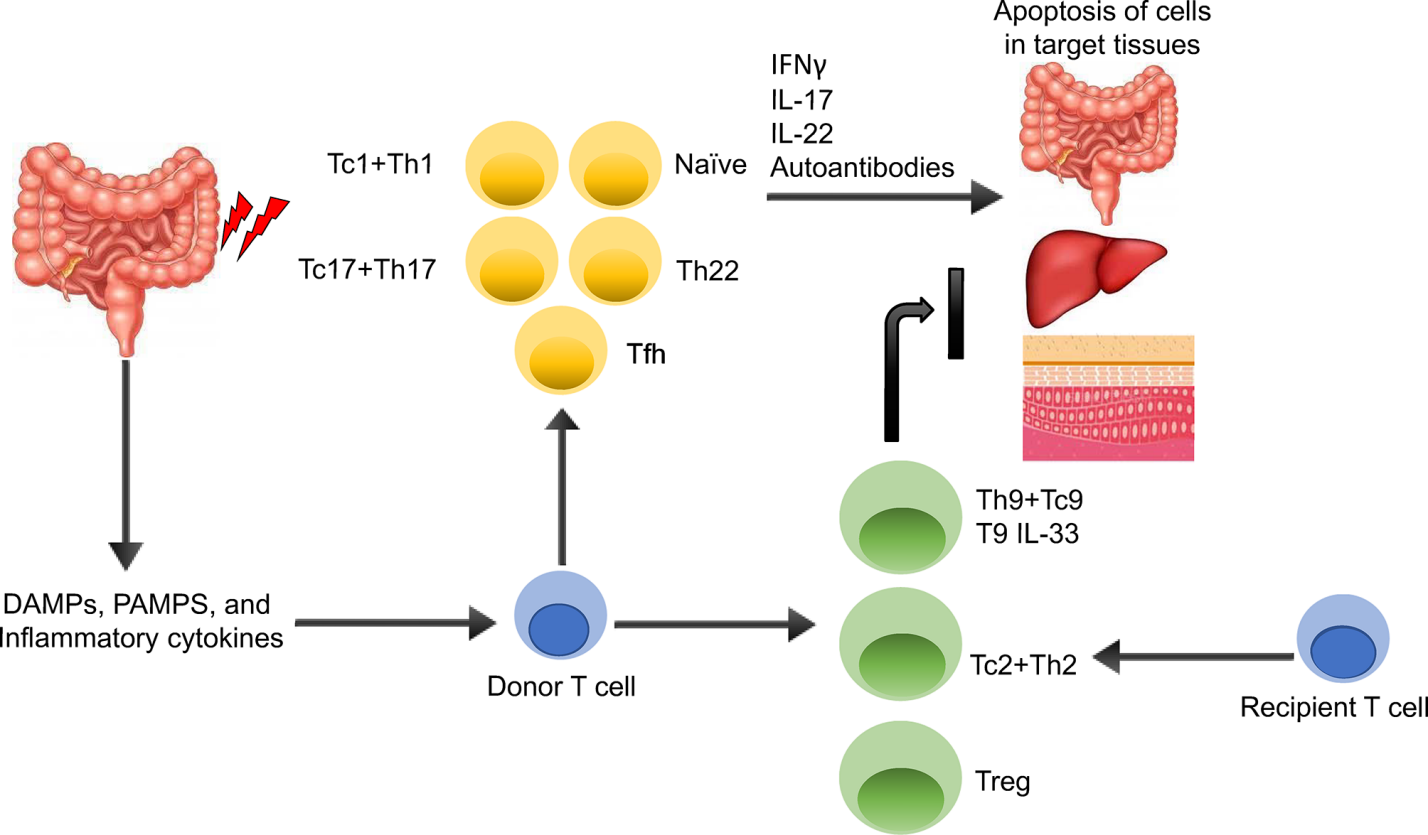
Overview of GVHD Pathogenesis. The gut and other issues are damaged during irradiation or chemotherapy, leading to the release of various DAMPs, PAMPs, and inflammatory cytokines. These DAMPs, PAMPs, and cytokines activate both host and donor antigen-presenting cells (APCs), which then activate the donor T cells. The APCs are also secreting various cytokines that promotes T cell differentiation toward different T cell subsets including T helper type 1 (Th1), T helper type 2 (Th2), T helper type 17 (Th17), T helper type 9 (Th9), and regulatory T cells (Tregs). Activated Th1 and Th17 T cells are able to secrete various pro-inflammatory cytokines including IFNγ, IL-17, IL-22 leading to apoptosis of cells in target tissues, mainly in the gut, liver, and skin, which can be alleviated by anti-inflammatory cytokine producing Th2, Th9 and Treg cells, such as IL-33-producing Th9.

**Figure 2** summarizes T cells subsets demonstrated or putative roles in GVHD/GVT. The gut and other tissues are damaged during irradiation and/or chemotherapy, leading to the release of various DAMPs/PAMPs, and inflammatory cytokines (22). These DAMPs, PAMPs, and cytokines activate both host and donor antigen-presenting cells (APCs), which then activate the donor T cells. The APCs are also secreting various cytokines that promotes T cell differentiation toward different T cell subsets including T helper type 1 (Th1), T helper type 2 (Th2), T helper type 17 (Th17), T helper type 9 (Th9), and regulatory T cells (Tregs). Activated T cells are able to secrete various pro-inflammatory cytokines including IFNγ, IL-17, IL-22 leading to cytolysis of cells in target tissues, mainly in the gut, liver, and skin, which can be alleviated by anti-inflammatory cytokine produced by Th2, Th9 and Treg cells, such as IL-33-producing Th9 (23).

### Naïve T Cells

T cell depletion previous to HCT and the use of T cell-depleting antibodies have been used resulting in a significant reduction in GVHD (24). Anti-T-lymphocyte Globulin (ATG) has been used to prevent GVHD in the conditioning regimen but also as treatment while the patient’s response is still unsatisfactory. In the steroid-refractory GVHD therapeutic setting, the response rate is reported to be 24%-41% using ATG and overall survival is poor (25). A recent phase 3 clinical trial aimed to take advantage of the benefits of T-cell depletion with respect to GVHD by using an anti-CD25 antibody (inolimomab) versus ATG, which found no difference in overall survival (26). Unfortunately, the experimental group suffered from issues with infection and relapse, common to T cell-depletion strategies leading to mortality in both arms of the study. The differences between naïve T cells and memory T cells have being investigated to determine which specific subsets of T cells were particularly inductive of pathological immune responses. Naïve T cells (T_N_) are CD45RA^+^CD62L^+^ antigen inexperienced cells with a diverse TCR repertoire (27, 28). Preclinical studies have supported the role of naïve T cells (T_N_) in inducing GVHD as opposed to central memory T cells (T_CM_) (29, 30). In allogeneic mouse models of HCT, it was determined that T_N_ caused more severe GVHD compared to that of T_CM_ and effector memory T cells (T_EM_) cells in isolation (29–35). It was also found in *in vitro* studies that CD8^+^ T_N_ were 5-20 times more likely to be specific for a minor histocompatibility antigen than T_M_ (36), supporting the role of this subset in the pathogenesis of GVHD disease. Concurrently, grafts in mice performed with memory cells retained GVT activity when challenged with malignancy (29, 34, 37). A recent phase II clinical trial applied these findings to humans. Naïve T cell depletion was used to reduce GVHD in acute leukemia patients (38). Naïve T cells were depleted from peripheral blood stem cells (PBSCs) using an iron-dextran bead conjugated to a monoclonal anti-CD45RA antibody. Thirty-five patients with acute leukemia or advanced myelodysplastic syndrome received T_N_-depleted HCT after myeloablative conditioning with 50 days of tacrolimus as immunosuppression. Durable engraftment was achieved in 34 out of 35 patients. Acute GVHD was not reduced in this trial. However, chronic GVHD, a secondary endpoint, was very low (9%) over the 2.5-year median follow up time. This method had other advantages when compared to historical controls, such as faster immune reconstitution compared to T-Cell Depleted (TCD) HCT, a 2-year disease free survival of 70% compared to 50% in TCD and 65% in T-cell-replete grafts, and a 28% relapse rate compared with 60% in TCD and 37% in T-cell replete grafts. These findings require confirmation in future randomized clinical trials.

### Th1 and Tc1

The early phase of aGVHD pathogenesis is predominantly mediated by Th1/Tc1 cells, with hyperproliferation and high cytotoxicity driving disease. These cells arise in response to the transplant-conditioning-induced cytokine storm and resultant release of DAMPs and PAMPs, and they can often be targeted by standard immunosuppression regimens, which focus on the inhibition of proliferation and NFAT-driven T cell signaling (39). CD4^+^ (Th) and CD8^+^ (Tc) T cells are stimulated to differentiate into the Th1/Tc1 together type 1 subtype when they receive T Cell Receptor (TCR) stimulation from their specific antigen, here allo-antigen, as well as co-stimulation from a variety of different pathways. IL-12, in its activation of STAT4 has been found to be a critical component of the Th1 pathway (40, 41). In the same way, CD8^+^ T cells are encouraged to differentiate into Tc1 cells by TCR activation, co-stimulation, and the cytokines IL-2 and IL-12 (42–44). Interferon gamma (IFNγ), a primary mediator of inflammation and tissue damage, is a primary product of activated Th1 cells (45). The defining Th1 transcription factor is T-box expressed in T cells (T-bet or Tbx21) (46). Similar to Th1’s, Tc1 cells also depend on T-bet as a transcription factor and are also induced by concurrent expression of eomesodermin (Eomes) (47).

Allogeneic donor Th1 and Tc1 cells have been shown in multiple experiments to induce both GVT and GVHD in mouse models and is classically thought to be the main propagator of GVHD. The IFNγ secreted by donor Th1 cells have been found to both encourage further Th1 cell differentiation and direct damage to the gut mucosa (48). Preclinical models have shown elevation of Th1-derived cytokines including tumor necrosis factor (TNF) and IFNγ in association with GVHD. The cytokines have also been implicated directly in target organ damage (48–51). T-bet is a crucial regulator of Th1 differentiation and IFNγ production, and T-bet^-/-^ T cells alleviate GVHD after adoptive transfer in both major and minor MHC mismatched mouse model (52). Blockade of Th1 and Th17 differentiation by targeting T-bet and RORgammat in mice ameliorates GVHD while surprisingly not decreasing GVT activity (53). As a transcription factor, a promising strategy is to target its downstream effectors for preventing GVHD instead of directly inhibiting T-bet. In human patients suffering from acute GVHD, Th1 cytokines are found in pathologic lesions, supporting the clinical relevance of this subset in the pathogenesis of GVHD (54, 55).

The Janus Kinases (JAKs) family members (JAK-1, -2, -3, Tyrosine kinase 2) and its downstream regulators signal transducers and activators of transcription (STAT) are crucial in the pathogenesis of GVHD (56). Different JAK inhibitors, such as JAK1/2-inhibitor ruxolitinib and JAK1-inhibitor itacitinib, have been developed and applied to prevent or treat aGVHD and cGVHD with different clinical indications. JAK1/2 antagonists can suppress Th1 and Th17 cell function, activation of antigen presenting cells (APCs), MHC expression and co-stimulatory signals through inhibition of STAT1 and STAT3 signaling pathways (57). The regulatory T cell function is retained by reserving IL-2-JAK3-STAT5 signaling pathway followed JAK1/2 inhibition. In 2019, ruxolitinib (Jakafi), a JAK1/2 inhibitor, was approved by the U.S. Food and Drug Administration to treat steroid-refractory aGVHD in adults and children age 12 years and older based on the randomized phase III trial (15). It was also recently approved for steroid-refractory cGVHD in the same population based on the randomized phase III trial (16). In recently published preclinical work, the JAK1/2-inhibitor baricitinib has shown to prevent GVHD by increasing Tregs *via* the JAK3 pathway (58).

### Th17 and Tc17

Th17 cells are the other major subtype of inflammatory T cell implicated in the pathogenesis of GVHD. Cytokines TFG-β1 and IL-6 ± IL-23 direct the differentiation of Th17 cells (59–63). The cells are defined by their expression of IL-17 and lack of expression of IL-4 and IFNγ (64, 65). Retinoic acid receptor-related orphan receptor gamma (RORγt) is the main transcription factor of the Th17 lineage (66). The role Th17 in the pathogenesis of aGVHD is complicated, but overall aGVHD appears to be primarily a Th1 but not Th17 process (67) while cGVHD is both a Th1 and Th17 process (68). Another Th17-like cytokine, IL-21 has also been shown in many preclinical models to induce aGVHD, either *via* knockout of IL-21 or inhibition of the IL-21 receptor (68–72). The role of Th17 cells have also been investigated in mouse models by way of their transcription factors. Multiple studies have found that the absence of both RORγt and Tbet greatly diminished the severity of aGVHD. In addition, absence of critical Th17 transcription factors led to a significant decrease in the frequency of IL-17A and TNF in subjects’ serum and pathogenic lesions (73). A third method to investigate the role of the Th17 subset in the pathogenesis of GVHD has been to target the cytokines that produced Th17. While likely not specific to Th17, IL-6 inhibition in mouse models was shown to significantly decrease aGVHD severity (74, 75). In addition, blockade of IL-23 was found to diminish aGVHD severity (76–78). Finally, allogeneic donor Th17 cells have been shown to be capable of inducing lethal GVHD in isolation, but they have also been shown to be unnecessary in doing so, as the Th1/Tc1 subset is also sufficient to do so in isolation (79, 80).

In patients suffering from aGVHD, the frequency of Th17 cells in peripheral blood was increased along with the frequency of IL-17 (81). As time progresses after transplantation, Th17/Tc17 cells may become a major driving force of GVHD, secreting proinflammatory cytokines, providing a cellular reservoir for effector alloimmune cells, and supporting the Tfh-driven immune response that characterizes cGVHD (39). Indeed, Th17 cells have been even more heavily implicated in cGVHD in humans. They have been found to be present in increased frequency in the blood of cGVHD patients (81) and mixed Th1/Th17 cells were found in histological examination of cGVHD skin lesions (82). In addition, CD146 and CCR5^+^CD146^+^ CD4 T cells are present in increased frequencies in humans suffering from aGVHD and cGVHD, and these cells have been shown to be skewed toward a mixed Th1/Th17 phenotype (83, 84). In a murine model experiment, the potential application of RORγt inhibition has been studied with TMP778. Treatment resulted in a significant decrease in the observed pathology, like a group treated with an anti-IL-17 antibody (84). Furthermore, KD025, was explored in a murine model of cGVHD, which demonstrated a significant reduction in the symptoms of disease. The same study also demonstrated that KD025 inhibition decreased the production of IL-21, IL-17, and IFNγ in the PBMCs of patients suffering from GVHD (85). To follow up on these findings, a phase II clinical trial investigating ROCK2 inhibition with belumosudil (KD025) in the treatment of SR-cGVHD (NCT02841995) showed overall response rates (ORR) (95% CI) with belumosudil 200 mg once daily, 200 mg twice daily, and 400 mg once daily of 65% (38% to 86%), 69% (41% to 89%), and 62% (38% to 82%), respectively. Responses were clinically meaningful, with a median duration of response of 35 weeks, and were associated with quality-of-life improvements and corticosteroid (CS) dose reductions (86). Furthermore, the ROCKstar study showed that belumosudil showed responses for cGVHD after 2 or more prior lines of therapy (87). Based on these findings, belumosudil was recently FDA approved for patients 12 years and older who have received 2 or more prior lines of therapy (88).

### Th22

Recently defined as a separate lineage from Th17 cells, Th22 cells were first described in the context of epidermis-infiltrating cells in individuals with inflammatory skin conditions that produced IL-22 and TNFα without producing IFNγ, IL-4, or IL-17 (89). Th22 cells have been shown to develop under the influence of IL-6, IL-23, IL-1β, and 6-formylindolo[3,2-B] carbazole (FICZ) *in vitro*, along with the tyrosine kinase inhibitor galunsertib. However, ideal conditions for differentiation of Th22 *in vitro* and *in vivo* have yet to be determined (90). RORγt has been established as the critical transcription factor for Th22 differentiation, while Tbet is an inhibitory transcription factor for this lineage (90). In contrast to the relatively well-established roles of Th1 and Th17 cells in the pathogenesis of GVHD, the role of Th22 cells and their trademark cytokine, IL-22, remains controversial. In murine models of aGVHD, approximately half the cytokine IL-22 was derived from Th22 cells (91). However, IL-22 has been associated with a protective effect on intestinal stem cells in an experiment that showed recipient deficiency in IL-22 led to more severe immune-mediated damage in the intestine (92). Simultaneously, it was demonstrated in a murine allo-HCT model that deficiency of IL-22 in donor T cells led to diminished aGVHD severity without inhibiting GVT (93). In line with this latter finding, exogenous injection of IL-22 into a murine model after allo-transplant was associated with increased aGVHD severity secondary to Th1 and Tc1 cell expansion, while diminishing Treg levels (94). However, the tissue protective functions of IL-22 can be decoupled from pro-inflammatory actions through structure-based design (95). Based on these findings, a study of IL-22 IgG2-Fc (F-652) along with corticosteroids for subjects with grade II-IV lower gastro-intestinal (GI) aGVHD has been conducted (NCT02406651). Preliminary results of the multicenter prospective phase 2 study showed the combination with corticosteroids was well tolerated and met primary efficacy endpoint (96). Based on these preliminary results, Genentech has sponsored an ongoing clinical trial investigating the use of IL-22Fc in addition to standard therapy for prophylaxis of aGVHD in patients undergoing allogeneic HCT (NCT04539470). Altogether, the action of IL-22 appears to depend on its source and location with donor IL-22 leading to increased aGVHD.

### Tfh

Tfh cell differentiation is a multi-step process that is initiated by dendritic cell priming of a naïve CD4^+^ cell (97). IL-6 is key to this priming process, and its signaling will increase the key transcription factor B-cell lymphoma 6 (Bcl6) in the maturing cell (98–101). Tfh secrete IL-21 as its lead cytokine (102). IL-2 acts as an inhibitor of the Tfh pathway (103, 104). While Tfh cells have not yet been investigated for their roles in aGVHD, donor Tfh cells have been shown to induce cGVHD *via* their secretion of IL-21. This cytokine leads to the proliferation of germinal centers, differentiation of plasma cells, and the production of auto-antibodies characteristic of cGVHD (105–107). Patients with active cGVHD had a significantly lower frequency of circulating Tfh compared with patients without cGVHD which was associated with higher CXCL13 plasma levels suggesting increased homing of Tfh to secondary lymphoid organs. Further, cTfh were skewed toward a Th2/Th17 phenotype in turn promoting B-cell immunoglobulin secretion and maturation (106).

### Trm

It was previously thought that T cells were exclusively found in the blood and secondary lymphoid organs at steady state. Recent observations suggest that the majority of memory T cells reside in human peripheral tissues, primarily located in the skin, gut, liver and lung. Increasing studies unraveled that tissue resident memory T (Trm) cells, representing a lineage of memory T cells, are thought to be contributors in the pathogenesis of GVHD. The Trm cells can be identified by specific markers like CD69 (108). Contribution from host T cells has been recognized recently. Pretransplant conditioning which typically consists of chemoimmunotherapeutic drugs and/or total body irradiation were thought to eliminate host T cells and therefore not play a role in GVHD, but new studies indicate that host T cells resident in peripheral tissues are highly resistant to depletion, even after high-intensity conditioning (109). In humans, host-derived Trm cells have been found in patients’ skin lesions before and after allo-HCT and showed distinct transcriptomic program with RUNX3 and galectin-3 as the phenotypic signatures for these cells as compared to blood T cells (110). Similarly, host T cells were found in all skin and colon from patients with aGVHD after allo-HCT. A subset of host-derived Trm cells is highly proliferative and can be directly activated by donor-derived monocytes. These Trm cells promote the development of GVHD through production of proinflammatory cytokines such as IFNγ and IL-17 (109). Skin Trm cells are HCT conditioning resistant and can be maintained during a long period of time with replenishing T cells rapidly acquiring Trm phenotype. The role of Trm cells in other GVHD target organs is also being explored in preclinical models as well as additional functional roles. For example, murine PSGL1^lo^CD4^+^ T cells from GVHD target tissues enhance B cell differentiation into plasma cells and production of autoantibodies *via* their PD-1 interaction with PD-L2 on B cells. Similar evidence was found from humanized GVHD target tissues. In addition, human PSGL1^lo^CD4^+^ T cells were apposed with memory B cells in the liver tissues of humanized mice and cGVHD patients (111). By creating three spatiotemporal T cell compartments in non-human primates, development of pathogenic Trm into donor CD8^+^ T cells after allo-HCT was observed. Results showed that by day 8 after transplant, donor T cells infiltrated into the GI tract and exhibit Trm hallmarks. The T cells displayed highly activated and cytotoxic phenotype driven by IL-15 and IL-21 signaling (112).

## Regulatory T Cells in GVHD

### Th2

Th2 cells mainly produce IL-4, IL-5, IL-10, and IL-13 (113). GATA-binding protein 3 (GATA3) was found to be the master transcription factor for Th2 cells (114). Tc2 cells overlap with Th2 cells in many ways, including their cytokine profile and transcription factor. However, they express both less IL-4 and GATA3 than Th2 cells (115, 116). While we placed Th2 cells under the “regulatory” section due to their protective role when adoptively transferred (117, 118), their overall role is still controversial as they have also been shown to be involved in the pathogenesis of GVHD of the skin and lungs at later stages (48, 119). IL-10-producing Th2 subset has been associated with decreased GVHD in animal models. Also, the natural protective effect of Th2 cells on the gut may prove beneficial for preventing severe gut GVHD (120), the most lethal location of the disease. Additionally, Th2 and Tc2 cells have been described to mediate significantly less severe GVHD compared to Th1 and Tc1 cells after adoptive cell transfer (117, 118). However, these cells concurrently have little to no ability to kill malignant leukemia cells *in vivo* (117, 118). No conclusive results can be drawn for an association between Th2/IL-4 and cGVHD.

Rapamycin resistant T cells (Trapa) hold promise in preventing GVHD in adoptive cell transfer. Trapa cells have the advantage of being more robust *in vivo* due to their increased frequency of the T central memory phenotype (Tcm). Rapamycin resistant T cells also have the advantage of proliferating to a greater degree compared to rapamycin sensitive cells once removed from rapamycin (121). These qualities have been exploited in both preclinical and clinical studies. *Ex vivo* murine Trapa cells polarized with IL-4 toward a Th2 phenotype differentiated into the Th2-type cell and was more effective at preventing GVHD and graft rejection than control Th2 cells (122). Rapamycin-resistance in T cells has also been shown to support Treg cell populations *in vivo* in the setting of transplant, denoting another potential avenue of rapamycin and rapamycin resistance to combat GVHD (123). A phase II clinical trial investigated Th2-skewed Trapa cells used as donor leukocyte infusion (DLI) after allo-HCT. Trapa cells showed a mix of Th2 and Th1 phenotype and cumulative incidence probability of aGVHD was 20% and 40% at days 100 and 180 post-HCT, respectively. Safety was demonstrated, as none of the patients experienced transplant-related mortality (124). However, there are no phase III Trapa DLI clinical trials in process.

### Th9

Th9 cells were shown to be a subset of CD4 cells unique from Th2 cells due to their significant IL-9 production and minimal IL-4 production (125). Characterization of this subset continued as transforming growth factor-beta was found to induce IL-9 expression in Th2 cells (126). A concurrent study similarly found that IL-4 along with TGF-beta led to an IL-9^+^ IL-10^+^ Foxp3^-^ phenotype (127). Eventually, PU.1 was deciphered to be a defining transcription factor of this unique subset (128). Recently, it was described that CD8^+^ cells could also differentiate into this IL-9-producing subset, representing Tc9 cells (129). Unlike the relatively straightforward role of Tregs, the function of Th9 and Tc9 cells in the context of immunomodulation is complicated, as they have been implicated in both pro-inflammatory and anti-inflammatory actions. It was suggested that Th9 cells may prevent GVHD in an experiment that showed in a murine allogeneic model, mice treated with a co-transfer of rapamycin resistant Th9 cells showed decreased donor CD8^+^ cell engraftment and decreased donor IFN-γ production (130). In addition, two studies suggested the importance of IL-9 specifically to immune-mediated limitation of tumor growth (131, 132).

Aside from the decreased IFN-γ with Th9 cell transfer, it was postulated that this subset may decrease GVHD through their expression of membrane-bound Stimulation-2 (ST2), the IL-33 receptor (23, 133). The IL-33/ST2 pathway has been shown to induce type 2 cytokine production, which is implicated in both supporting tissue repair and maladaptive allergic responses (134). Elevated levels of soluble ST2 (sST2), the decoy receptor, was found to be a risk factor for severe GVHD (135). Using an anti-sST2 antibody GVHD severity could be reduced. This treatment also simultaneously maintained membrane-bound ST2 expression on T cells, increasing the ratio of ST2 to sST2, as well as maintaining GVT. This combination of findings pointed to the inverse relationship between the two related receptors (136).

It has previously been described that Th2 cells express ST2 (137, 138) and that its ST2 expression is increased upon exposure to TGF-beta and IL-33. Further, it was found that IL-33 and TGF-beta treatment increased the expression of IL-9 by Th2 cells (139). With these existing data, it was hypothesized that cells polarized under Th9/Tc9 conditions with the addition of IL-33 would exhibit even greater anti-GVHD effect with maintenance of GVT (23). Indeed, this was found to be the case. Furthermore, supporting evidence was found for the mechanism of the T9_IL-33_ subset’s mechanism of GVHD prevention in that this subset expressed significantly more amphiregulin (AREG) on its surface than other subsets. Further research on the new T9 cell subset found that cholesterol blockade in Th9 cells with beta-cyclodextrin led to significantly increased IL-9 production as well as increased tumor killing in both a melanoma model and a metastatic lung tumor model (140). The combination of IL-33 with an anti-cholesterol agent to further enhance the desirable phenotypic characteristics of this subset is an exciting potential avenue of research that could be applied in the near future to combat GVHD while maintaining GVT. Of note, like aGVHD, sST2 is elevated in patients with cGVHD (141). However, the role of the ST2/IL-33 pathway in preclinical model of cGVHD is still under study.

### Amphiregulin (AREG)-Expressing T Cell

AREG, a member of Epidermal Growth Factor (EGF) family, binds to EGF receptor and promote the proliferation of normal and malignant epithelial cells, fibroblasts and keratinocytes. Deficiency of AREG in mice showed slower clearance of helminth parasite, Trichuris muris, which was driven by Th2-biased responses (142). Recent study has revealed that IL-33 *via* its receptor ST2 enhances the production of AREG from ST2^hi^ memory T helper 2 (Th2) subset, and directly involved in the reprogramming eosinophils to an inflammatory state with a boost production of osteopontin, a key profibrotic immunomodulatory protein which hence contribute to establishing of lung fibrosis (143). As described above, T9_IL-33_ surface expressed amphiregulin (AREG) contributes to its GVHD prevention. Furthermore, AREG was found to be essential to the anti-GVHD effect of T9_IL-33_ cells co-cultured with allogeneic colonic epithelial cells, as AREG blockade significantly increased epithelial cell damage. It was also found that AREG did not cause suppression of effector T cell subsets, explaining the ability of T9_IL-33_ cells to simultaneously inhibit GVHD and maintain GVT (23). In further support of AREG’s lack of suppressive activity, AREG was previously found to be inconsequential in the suppressive activity of Tregs *via* genetic ablation (144). Another recent work has revealed that *ex vivo* IL-33-stimulated Tregs (termed as Treg_IL-33_) expressed higher AREG and displayed stronger immunosuppression. Adoptive transfer of Treg_IL-33_ led to a marked improvement of GVHD prevention compared to either naïve control Tregs or IL-23/IL-17-stimulated Treg_IL-33_. Consistently, blocking AREG with neutralizing antibody *in vivo* abolished the immunosuppression function of Treg_IL-33_, which collectively suggest a critical role for AREG in IL-33/Treg-mediated GVHD control (145).

### Classical Regulatory T Cells (Tregs)

*In vivo*, Tregs have been shown to develop under the influence of IL-2, IL-15, and TGF-β with FOXP3 as the most critical transcription factor (146–149). Tregs have an extensive experimental history with respect to GVHD, as recently reviewed (150). As predicted by their inherent biology of immunomodulation and self-tolerance, Treg populations have been shown to be decreased during GVHD, allowing for alloreactive T cells to exert their effect (151). Tregs that are FOXP3 negative are known as Type 1 regulatory (Tr1) cells (152). Tr1 cells were characterized as being generated due to alloantigen stimulation by a recipient dendritic cell as well as being stimulated by IL-27. The source of IL-27 is mainly donor macrophages in the context of allo-HCT. In conjunction with this finding, it was determined in this work that IL-6 inhibition increases the proliferation of Tr1 by increasing T cell sensitivity to IL-27 (153). Supporting the inhibitory role of Tregs in the pathogenesis of aGVHD, Tr1 deficiency has been found to exacerbate aGVHD in mouse models (153).

Naturally occurring Tregs (nTregs) have been studied for over a decade in mouse models investigating their ability to prevent aGVHD with nTreg transfer (154, 155). Recipient Treg populations have been expanded before allo-transplant in mice using tumor necrosis factor receptor-2 agonists, leading to prolonged survival and decreased aGVHD (156). Chimeric antigen receptor therapy has been applied to Tregs as well. An alloantigen (HLA-A2) specific CAR was created and applied to Tregs, thus creating an alloantigen-specific human Treg phenotype. In murine models, these CAR-T cells demonstrated superior xenogeneic GVHD prevention caused by HLA-A2^+^ T cells compared to Treg cells expressing an irrelevant CAR (157).

In human studies, Treg cells have become the front-runner in the use of cell transfer to treat GVHD. Naturally occurring Tregs (nTregs) hold significant promise as a therapy, but nTreg use in clinic has been hindered by a limited amount of Tregs in the peripheral blood (1-2%) (158, 159) and contamination of nTregs with CD25^+^ T-effector or T memory cells (159–161). However, good-manufacturing practice, large-scale *ex-vivo* expansion of Tregs has been demonstrated (159). And despite these limitations, human clinical trials using Tregs have shown promising results. A phase one and dose escalation study with umbilical cord-derived nTreg cells in the prevention of GVHD was encouraging for this style of therapy in the future (162, 163). Another trial investigated the effect of early infusion of freshly sorted Tregs followed by conventional T cells (Tcons) on immune reconstitution and GVHD after haplo-identical HCT. Results of this trial showed promise for GVHD prevention, immune reconstitution, preserved GVT, and resistance to opportunistic infections (164). It is worth noticing that the role of Tregs in cGVHD pathogenesis is controversial (165). Both donor and recipient derived Tregs are known to use TGFβ as the effector of suppression in several models. In contrast to its protective role in aGVHD, Treg-produced TGFβ may exacerbate cGVHD since TGFβ can result in fibrosis of organs such as the skin and lung (120).

In the ALT-TEN trial, patients underwent haplo-identical T-cell depleted HCT combined with IL-10 pretreated T cells. The IL-10 treated cells contained Tr1 cells and T memory cells. The results demonstrated the feasibility of using Tr1 cells as a treatment for immune-mediated disorders such as aGVHD (166). The inhibitory role of IL-6 on Treg and Tr1 expansion has been explored in a phase I/II clinical trial as a potential therapeutic target for aGVHD. Anti-IL-6 tocilizumab was used in a single dose before allo-matched HCT, which showed low incidence of aGVHD with treatment, and called for further study of this method in GVHD prophylaxis (167). However, a more recent randomized phase 3 trial evaluating the addition of tocilizumab to cyclosporin and methotrexate for aGVHD prophylaxis, did not show statistically significant reduction in grade II-IV aGVHD or long-term survival (168).

## T Cells Inducers of GVT

Donor grafts-derived allogeneic immune cells, particularly the T cells, recognize and eradicate leukemic cells *via* GVT reactivity, which hence could harness the power for high-risk hematological malignancies such as acute myeloid leukemia (AML) and multiple myeloma (MM). However, the normal tissues of the recipient will also be recognized and attacked by these cells also attack host normal tissues by GVHD (169). Separation of GVT reactivity from GVHD reaction is a necessary step for improving allo-HCT outcomes. Previous study indicated that Th9 cells, a unique subset of CD4^+^ T cell that produce the pleiotropic cytokine IL-9 and boost antitumor immune responses *in vivo via* CD8^+^ CTL-mediated antitumor immunity (131). Further study revealed that IL-9–produced CD8^+^ T (Tc9) cells generated various cytokines and showed less cytolytic activity *in vitro* but surprisingly elicited enhanced antitumor responses against advanced tumors in OT-I/B16-OVA and Pmel-1/B16 melanoma models (170). As proof of principle of better antitumoral activity, human chimeric antigen receptor (CAR) T cells polarized and expanded under a Th9-culture condition (T9 CAR-T) showed enhanced antitumor activity against established tumors compared to IL2-polarized (T1) cells. T9 CAR-T cells secrete IL9 but little IFN-γ, express central memory phenotype and lower levels of exhaustion markers and display robust proliferative capacity (171). In allo-HCT settings, T9 cells activated with IL-33 during *in vitro* differentiation boosted their ST2 expression and IL-9 production. Adoptive cell transfer (ACT) of IL-33 activated T9 cells (T9_IL-33_) decreased GVHD severity and increased GVT activity *via* two distinct mechanisms: decrease of fatal immunity by amphiregulin expression and increase of antileukemic activity *via* CD8α expression (23).

## Novel GVHD Treatments Based on Fundamental T Cell Biology

### Cellular Therapy

Recent reviews have summarized the scope of cellular therapies to treat GVHD (172, 173). **Table 1** list potential T-cells based cellular therapies at different stages of development. Cellular therapies are likely to expand their scope in patients with diverse diseases (174), although delivery of such “live” drugs are not easily scalable (175). Fortunately, in parallel strikes have been made in GVHD treatment with classical drugs as summarized below and in **Table 2**.

**Table 1:** Summary of Cellular Therapies for GVHD based on T cells subsets.

Treatment	Status
Naïve T cell depletion	Completed Phase II
Trapa DLI	Completed Phase II
Th9/TC9	Preclinical
HLA-A2 CART Treg	Preclinical
nTreg	Completed Phase I
Tr1 Expansion	Completed Phase II

**Table 2:** Summary of recent novel small molecule treatments for GVHD.

Treatment	Mechanism	Status	Trials (examples)
**Ibrutinib**	BTK/ITK inhibition	Completed Phase I/II Phase III ongoing	
**Ruxolitinib**	JAK inhibition	Completed Phase II/III for steroid-refractory aGVHD & cGVHD	
**Itacitinib**	JAK inhibition	Completed Phase I, Phase III for steroid naïve patients	INCB039110
**TMP778**	RORγt inhibition	Preclinical	
**KD025**	RORγt inhibition	Phase II	NCT02841995
**Tocilizumab**	Anti-IL-6	Completed Phase I/II/III	
**Brentuximab Vedotin**	CD30 conjugated Ab	Completed Phase I	
**Vedolizumab**	Integrin inhibition	Halted Phase II	NCT02993783
**Natalizumab**	Integrin inhibition	Phase II	NCT02176031, NCT02133924

### Small Molecules Inhibitors

#### ITK Inhibitors

Ibrutinib is an Interleukin-2-inducible T-cell kinase (ITK) and Bruton tyrosine kinase (BTK) inhibitor that hinders the survival of reactive T-cells, and B cells, respectively (176, 177). In mice, transplant of bone marrow deficient in ITK and BTK showed the importance of these molecules in the pathogenesis of cGVHD, as the transplanted mice did not experience cGVHD (25). Concurrently, mice treated with ibrutinib experienced less severe cGVHD (178). In a phase I/II study for patients with SR-cGVHD, ibrutinib was shown to significantly improve symptoms in most patients, as well as decrease the frequency of chemotactic and fibrotic factors in patients’ blood (179). A significant number of adverse events (AEs) including grade ≥ 3 infectious complications were seen; however the safety profile was deemed acceptable as the AEs were similar to those observed in cGVHD patients treated with concomitant steroids (179). These studies led to the first ever drug in cGVHD to obtain the FDA breakthrough denomination.

#### JAK Inhibitors

Janus kinases (JAKs) are tyrosine kinases that mediate cytokine-signaling in T cells, propagating survival and differentiation signals (180). The activation of a JAK leads to phosphorylation of signal transducers and activators of transcription (STATs) (181). JAK signaling has also been associated with dendritic cell function, thus amplifying this pathway’s potential importance in GVHD (182, 183).

In mouse models, JAK1/2 blockade with ruxolitinib has displayed decreased IFNy Receptor (IFNyR) receptor signaling, leading to reduced severity of GVHD and preserved GVT (184, 185). In addition, JAK1/2 inhibition in mouse models led to increased frequency of Tregs and decreased frequency of inflammatory cytokines in association with the decreased severity of aGVHD (181).

Following up on findings in murine models, a preliminary trial of 6 human patients with SR-GVHD treated with ruxolitinib showed an improvement in symptoms and similar reduction of the frequency of inflammatory cytokines in peripheral blood (181). In addition, a large multicenter retrospective survey of patients who had received ruxolitinib for steroid-refractory GVHD suggested that ruxolitinib had significant efficacy (186). The results of the phase III randomized clinical trials have recently been published with an overall response of 62% in the ruxolitinib group vs. 39% in the control group (P<0.001) in steroid-refractory aGVHD (15), and an overall response of 50% in the ruxolitinib group vs. 26% in the control group (P<0.001) in steroid refractory cGVHD (16), respectively. Ruxolitinib is now the second drug to get the FDA breakthrough denomination for both cGVHD and aGVHD.

A specific blockade of JAK1 was explored in a phase I trial with itacitinib (INCB039110), which showed responses rates of 64.7% and 88.3% for steroid refractory and treatment naïve disease, respectively (187). Similar AEs were seen with this drug as with ruxolininib, including cytopenia and CMV reactivations. However, itacitinib missed the mark in phase III when given in combination with corticosteroids in patients with treatment-naïve aGVHD.

#### RORγt Inhibitors

##### TMP778

As mentioned above, one of the RORγt transcription factor small molecule inhibitors, TMP778, has showed promise in a GVHD murine model similar to an anti-IL-17 antibody (84). However, global inhibition of a transcription factor is generally too toxic to implement in clinic ad alternative have been found such as ROCK2 inhibitors upstream of transcription factors.

##### ROCK2 Inhibitors

Belumosudil (KD025) is a serine-threonine kinase inhibiting ROCK2 that rebalances the immune system in GVHD by downregulating pro-inflammatory Th17 cells and increasing Tregs, also acting on JAK2/JAK3 and STAT3 (85). Further, ROCK2 is an intracellular integrator of profibrotic signals. Excellent responses were seen in the phase II clinical trials (86) (87) and belumosudil was FDA approved for cGVHD patients who are received 2 prior lines of therapy as mentioned above (88).

### Anti-Cytokines

#### Anti- IL6

The addition of a humanized anti-IL-6R mAb (Tocilizumab) to standard GVHD prophylaxis has shown in promise in reducing the incidence of aGVHD in a prospective phase I/II clinical study (167). The phase III double-blinded study of the addition of Tocilizumab vs. Placebo to cyclosporin/methotrexate GVHD Prophylaxis after HLA-Matched allo-HCT failed to meet the primary endpoint (168).

### Conjugated Antibodies (Ab)

#### Anti-CD30 Ab

Higher frequency of CD30^+^ CD8 T cells, plasma soluble CD30, and CD30+ lymphocytes have been demonstrated in the intestinal lesions of aGVHD patients (188). This led to the proposal of using the anti-CD30 monomethyl auristatin E (MMAE) conjugate for use in GVHD. A phase I trial for patients with SR-aGVHD showed significant toxicity associated with this drug, including neutropenic sepsis leading to death along with other grade III toxicities of headache, hypoxia, ileus, and elevated bilirubin (189).

#### Anti-Integrins Abs

Blockade of alpha4beta7 in the gut has been used effectively for inflammatory bowel disease as it disallows effector T cells from being trafficked to the area of inflammation. Natalizumab is one such example that has been used for autoimmune diseases such as Crohn’s, however, its lack of specificity gives it the associated risk of progressive multifocal leukoencephalopathy (PML) (190). Two phase II clinical trials are currently investigating the effectiveness of natalizumab for GVHD (NCT02176031 and NCT02133924) (14). Vedolizumab represents an example of a monoclonal antibody that is specific to the gut, as it inhibits alpha4beta7 integrin’s interaction with MAdCAM-1 and therefore carries significantly lower risk for the serious adverse outcome of PML (191–193). Similarly, this integrin has been shown to be important in the pathogenesis of intestinal GVHD (184). A recent case series of 6 patients explored the use of vedolizumab for the treatment of steroid-refractory intestinal GVHD. Patients treated with vedolizumab almost universally achieved remarkable improvement in gastrointestinal GVHD symptoms, in some cases having symptoms reduced from grade IV to grade I or absent (194). However, a phase II clinical trial, NCT02993783, to evaluate the safety and efficacy of this treatment was recently terminated due to lack of efficacy. Indeed, ORR at day 28 was 50% in patients treated at 300 mg (n = 8) and 22% in patients treated at 600 mg (n = 9); 12% and 0% of patients, respectively, achieved complete response (CR). Thus, higher the dose the less efficacious vedolizumab was. Due to its mechanism of action of blocking T cell migration to the intestine, it is likely that there cannot be an effect when GVHD is already full-blown and T cells in the gut. However, it is possible that preventing effector T cells migration to the GI tract may be beneficial at early stages of GVHD or as GVHD prophylaxis.

## Conclusion

Acute and chronic GVHD remain severe and common complications of hematopoietic stem cell transplant. Prevention and treatment of these diseases remain a critical frontier in transplant medicine. New understandings of T cell biology have led to novel treatments with a variety of targets and fundamental mechanisms. The plethora of recent human clinical trials as well as the exciting preclinical experiments have suggested the real possibility of a significant breakthrough for HSCT patients in the near future.

## Author Contributions

HJ, DF, and SP conceived and wrote the manuscript. All authors contributed to critical revisions of the manuscript. All authors contributed to the article and approved the submitted version.

## Funding

The National Cancer Institute (R01 CA168814, and U01 CA232491), and the National Heart Lung and Blood Institute R21-HL139934 to SP, and Medical University of South Carolina Smartstate funds for the Sally Abney Rose Endowment to SP.

## Conflict of Interest

SP holds a patent on “Biomarkers and assays to detect chronic graft versus host disease” (U.S. Patent #10,571,478 B2).

The remaining authors declare that the research was conducted in the absence of any commercial or financial relationships that could be construed as a potential conflict of interest.

## Publisher’s Note

All claims expressed in this article are solely those of the authors and do not necessarily represent those of their affiliated organizations, or those of the publisher, the editors and the reviewers. Any product that may be evaluated in this article, or claim that may be made by its manufacturer, is not guaranteed or endorsed by the publisher.
